# Recombining Low Homology, Functionally Rich Regions of Bacterial Subtilisins by Combinatorial Fragment Exchange

**DOI:** 10.1371/journal.pone.0024319

**Published:** 2011-09-07

**Authors:** D. Dafydd Jones

**Affiliations:** 1 School of Biosciences, Cardiff University, Cardiff, United Kingdom; 2 Novozymes A/S, Bagsværd, Denmark; National Institute for Medical Research, Medical Research Council, United Kingdom

## Abstract

Combinatorial fragment exchange was utilised to recombine key structural and functional low homology regions of bacilli subtilisins to generate new active hybrid proteases with altered substrate profiles. Up to six different regions comprising mostly of loop residues from the commercially important subtilisin Savinase were exchanged with the structurally equivalent regions of six other subtilisins. The six additional subtilisins derive from diverse origins and included thermophilic and intracellular subtilisins as well as other academically and commercially relevant subtilisins. Savinase was largely tolerant to fragment exchange; rational replacement of all six regions with 5 of 6 donating subtilisin sequences preserved activity, albeit reduced compared to Savinase. A combinatorial approach was used to generate hybrid Savinase variants in which the sequences derived from all seven subtilisins at each region were recombined to generate new region combinations. Variants with different substrate profiles and with greater apparent activity compared to Savinase and the rational fragment exchange variants were generated with the substrate profile exhibited by variants dependent on the sequence combination at each region.

## Introduction

Diversity among genes that code for proteins with the same core bioactivity (e.g. proteolysis) and protein fold (e.g. subtilisin-like) has been generated in nature over billions of years, and reflects both optimisation due to environmental selection pressures and neutral drift [1], [2], [3], [4], [5]. It is relatively common for proteins sharing a similar protein fold and bioactivity to display a high degree of diversity at both the amino acid and DNA level [6], [7]. However, these sequence and hence fine structural differences are important for defining the distinct bioactivity of each protein.

The subtilisins family of serine proteases are one such group of proteins. They play a variety of important biological roles that range from highly precise prohormone activating enzymes [8] to non-specific secreted bacterial digestive proteases [9] to intracellular protein-processing [10]. The bacilli subtilisins have been the focus of particular attention. Their generally broad substrate specificity and robustness to harsh environments coupled with further enhancement through both rational protein engineering [11], [12] and directed evolution [13], [14] has resulted in their exploitation by industry for a variety of applications [15], such as an active ingredient in laundry detergents. The bacilli subtilisins such as Savinase, BPN', subtilisin E and Subtilisin Carlsberg (Alcalase) have also proved important models for understanding the protein-structure-folding relationship [16], [17], [18], [19], [20], [21].

Specific regions of subtilisins are known to play important roles that define properties central to bioactivity [9], such as catalysis, substrate recognition, metal ion binding and structural integrity. It may therefore prove fruitful to interchange these functionally rich elements between the subtilisins. While traditional site-directed/cassette mutagenesis approaches allow limited region exchanges to be achieved in a low throughput manner [22], [23], [24], a combinatorial approach will allow sampling of multiple regions in different combinations from highly diverse sequences derived from numerous homologues. Such an approach expands the sequence set sampled and thus improves the chance of generating a protein with novel and useful properties. It also overcomes our current limitation to successfully predict the exact outcome of region exchange, which becomes further complex when multiple regions with sequences of varying length and character that interact in cooperative fashion are sampled. Directed evolution homologous recombination approaches [25], [26], [27] have been used to mix closely related protein homologues but the requirement of relatively high sequence identity restricts their use with respect to sampling more distant and functionally distinct relatives. Approaches such as SCHEMA [28] attempt to identify crossover positions that are least likely to result in structural disruption but does not focus on functionally rich regions of proteins.

**Figure 1:**
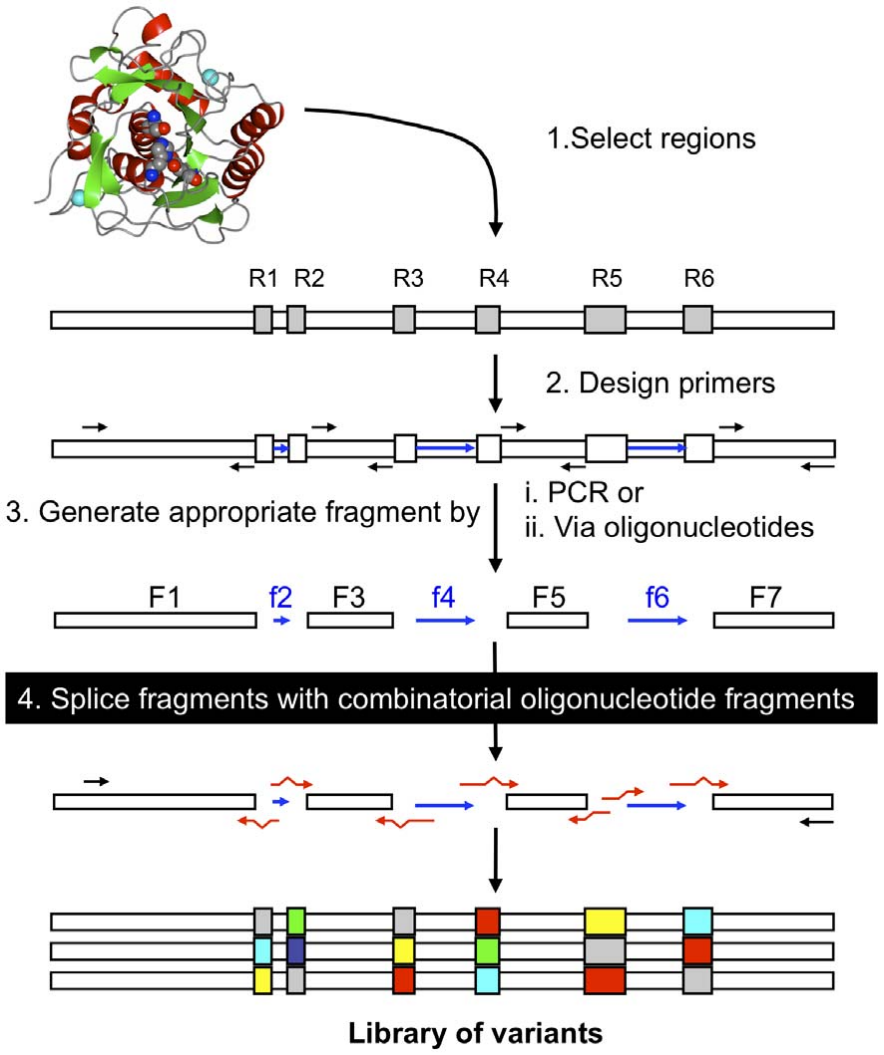
Combinatorial fragment exchange. (1) Regions known to play an important role in the protein structure-function relationship are chosen and defined based on alignment of known structures or homology models. (2) Appropriate oligonucleotide primers are designed to fragment the gene encoding the core protein scaffold based on the structural alignment determined in (1). (3) The oligonucleotides generated in (2) are used to fragment the core gene. (4) Oligonucleotides encoding the selected regions from the selected donor sequence elements (red arrows) are used to splice together the core gene fragments generated either by PCR (white blocks; labelled F*n*) or bridging oligonucleotides (blue arrows; labelled f*n*). This generates the library of variants with different combinations of sequences at each of the selected regions. The example given in the figure represents the fragmentation and reassembly approach used in this study.

To overcome some of the limitations of current approaches, a combinatorial fragment exchange (ComFrEx) method is utilised here (Figure 1). In an approach analogous to antibody complementary determining region engineering [29] multiple selected regions originating from a range of different subtilisins are recombined within the context of a common core backbone donated by Savinase to generate new protein variants. Up to 6 functionally rich regions of Savinase were replaced with the corresponding regions from 6 different bacilli subtilisins. Savinase was largely tolerant to a wide variety of different region combinations despite a sequence identity of ≤60% with the other subtilisins. The substrate specificity of the hybrid variants varied and depended on the sequence combinations at each region.

## Results and Discussion

### Selection of regions to be exchanged

To allow effective shuffling of important functional regions of subtilisins, a combinatorial fragment exchange approach was employed (Figure 1). Such an approach will allow sequence homology-independent recombination of multiple regions of a variety of different sequences originating from homologues with varying degrees of sequence identity to the core target protein. Loop regions for example can play pivotal roles in defining functional differences between protein homologues yet their sequence, length and structure can vary dramatically [22], [23]. Furthermore, loop regions are more adaptable than regular secondary structure and thus accommodate replacement more readily. Thus a loop region, which can be precisely defined by analysis of a protein structure or model, may represent a recombination unit. Precise structural alignments (using known 3D structures or homology models) allow the exact sequence region to be exchanged thus overcoming sequence anomalies due to insertions or deletions or inherent low sequence homology. Inclusion of insertion and deletions will allow a broader sampling of conformational space than that accessed by substitutions alone; indel mutation have been found to be beneficial in altering properties such as substrate specificity [30].

**Figure 2:**
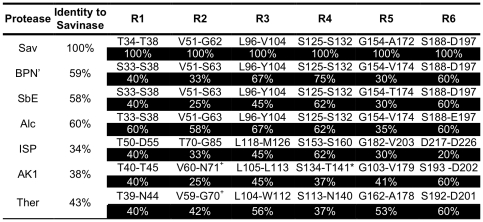
Sequence identity and region definition of chosen subtilisins. Region marked with a + contains an additional calcium binding site and the S49D mutation to Sav core. The regions marked with * a Cys-X-Cys disulphide bond.

**Figure 3:**
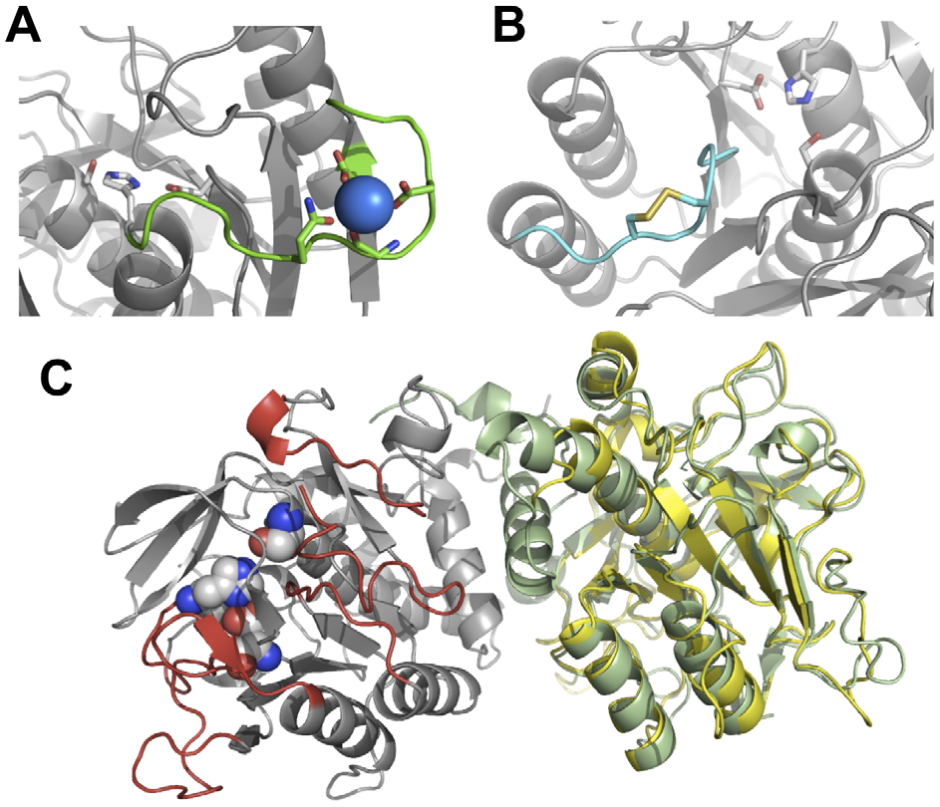
Structural features of selected donor subtilisins. (A) The additional calcium binding site observed in R2 of Ther and AK1. Calcium ion is shown as blue sphere, R2 is green with calcium coordinating residues shown as sticks. The catalytic triad is shown as grey sticks. Diagram created using PDB file 1THM [41]. (B) The Cys-X-Cys disulphide bridge in the R4 region of AK1 (PDB code 1DBI [31]). R4 is coloured cyan, with the disulphide bridge shown as sticks. The catalytic triad is shown as grey sticks. (C) Structure of ISP from *B. clausii* (PDB code 2X8J [33]). The two protomers are coloured grey and green respectively. The catalytic triad (space-fill) and the 6 selected regions (coloured red) are shown on the left, grey protomer. The homology model of ISP generated as described in the material and methods is coloured yellow and overlaid on the right, green protomer of the experimentally determined structure of ISP.

Seven different subtilisins were chosen to act as sequence donors (Figure 2). Sav was chosen as the core protein (providing the majority of the backbone and supplying the signal sequence and prodomain) due to its general robustness, as highlighted by its widespread usage in different commercial applications far removed from its normal biological context [15]. Three other commonly studied and utilised bacterial subtilisins were also chosen to contribute diversity; Alcalase® (also known as Subtilisin Carlsberg; Alc), BPN' (BPN), and Subtilisin E (SbE). Two subtilisins derived from thermophiles were also included; Thermitase (Ther) and *Bacillus* Ak.1 (AK1). They also contain a novel calcium binding site that is thought to contribute stability (Figure 3A). Furthermore, AK1 has a relatively uncommon Cys-X-Cys disulphide bridge separated by only one residue (Figure 3B) that contributes to thermostability and formation of the substrate binding [31], [32]. While the detailed 3D structure of the above selected subtilisins were known, there was no structure available before the commencement of this work for the final sequence contributor, the intracellular subtilisin protease (ISP). The ISPs are the only members of the bacterial subtilisins that function within the cell and have sequence features that differ from their secreted relatives, including the absence of the classical prodomain [33]. The sequence identity of ISP to Sav was also the lowest amongst the selected homologues (Figure 2). Therefore, for the purposes of this work a structural model of an ISP from *B. clausii* was generated (Methods S1). Comparison of the homology model with the recently determined 3D structure of the ISP [33] (Figure 3C) revealed a Cα r.m.s.d of 0.87 Å, confirming the model provided an excellent template for region selection. Thus, homology models can be used as a realistic alternative in the absence of a protein structure. All the structures of the selected subtilisins aligned closely to Sav, with the r.m.s.d. over the backbone ranging from 1 Å (BPN) to 2 Å (AK1).

**Figure 4:**
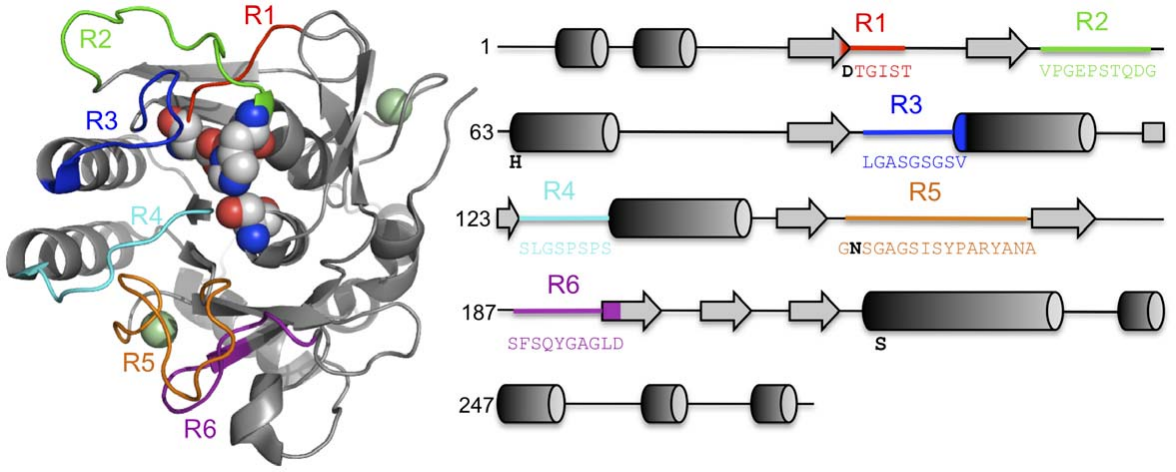
Selected Sav regions for ComFrEx. The structure of Sav (1SVN; [37] with each of the six regions highlighted as shown in the top panel of figure. The catalytic triad is shown as space-fill and calcium ions shown as green spheres. The right hand panel defines each of the regions in terms of their placement within Sav primary and secondary structure. The sequence of each region is shown and coloured as indicated in the diagram. Letters in bold and coloured black indicate residues contributing to the catalytic triad and anoxyion hole.

Six regions were chosen for ComFrEx (Figure 4 and Figure 2) on the basis that they are (a) involved in substrate binding, (b) contribute to metal ion binding, (c) involved in catalysis, (d) directly interact with regions mentioned previously, (e) mutations in these regions have shown beneficial effects, (f) all of the former. R1 is comprised of a loop that lies immediately after the conserved catalytic triad residue D32 (Figure 4) and contributes to the formation of the S2 substrate binding pocket that defines the preference of substrate at the P2 position [9]. R2 is a loop that directly interacts with R1 and lies immediately before the conserved catalytic triad residue, His64 (Figure 4). It also contains the third calcium binding site in Ther and AK1 (Figure 3A). Three of the coordinating groups are in the R2 region, with the fourth lying in the β-strand immediately before R2. To promote calcium binding, R2 of Ther and AK1 incorporated the S49D mutation to introduce the fourth calcium-coordinating group. R3 and R4 form part of the substrate-binding pocket and are especially important for determining preference at the P4 position of the substrate [9]. R4 of AK1 contains the Cys-X-Cys disulphide bridge (Figure 3B). R4 along with R5 contribute to the formation of a large cleft that binds the P1 position of the substrate [9]. R5 also contains Asn155, the residue that forms the anoxyion hole (Figure 4). R5 together with R6 contributes to the formation of low affinity metal binding sites. R6 also directly interacts with R5 and is involved in substrate binding.

### Generation and analysis of rational variants

**Figure 5:**
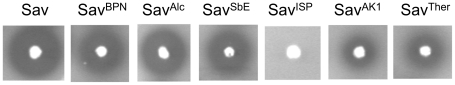
Activity of rationally constructed Sav-hybrid variants. The donor fragments at each of the 6 regions derives from one of the subtilisins, which is indicated in superscript in the figure. An active variant is indicated by the production of a clearing zone or halo around the *B. subtilis* colony due to digestion of casein embedded in the agar growth medium.

To assess the general impact of a rational fragment exchange strategy and the tolerance of the Sav core to the replacement of the six regions, variants were constructed whereby all 6 regions were replaced with the equivalent sequences from one of the chosen subtilisins. All the rational variants apart from Sav^ISP^ (Sav with regions R1 to R6 replaced by the ISP sequences) when expressed in *B. subtilus* generated significant clearing zones indicating the production of an active protease (Figure 5A). Therefore, it appears that Sav can tolerate the incorporation of all the regions from the chosen subtilisins despite sequence identities dropping as low as 25% (Figure 2).

The proteolytic activity of the cell cultures producing each of the variants was also assessed using two colourmetric *p*-nitroanilide (pNA) peptide substrates, Succinyl-Phe-Ala-Ala-Phe-pNA (FAAF) and Succinyl-Ala-Ala-Pro-Phe-pNA (AAPF). All the rational variants, except Sav^ISP^ exhibited activity towards FAAF but to various levels (Table 1). The observed activity of the reconstituted Sav variant was ∼20 fold higher than the next most active variant, Sav^BPN^. The observed activity of the cell cultures for Sav^AK1^ and Sav^Ther^ were the lowest of all suggesting the Sav core can barely tolerate all the 6 regions being occupied by sequences donated by these thermophilic subtilisins. The observed decrease in activity for each of the rational variants may be due to disruption of key catalytic processes or, more likely, destabilisation of tertiary structure leading to lower amounts of active folded protease in the culture medium. However, it cannot be ruled out that the mutations introduced into Sav decreased compatibility with the prodomain, which may affect folding and/or autocatalytic processing (which acts as an inhibitor if not removed).

**Table 1:** Activity of rationally constructed variants

Variant^a^	Activity			
	Halos	FAAF^b^(µM/min)	AAPF^c^(µM/min)	FAAF:AAPF ratio^d^
Savinase	Yes	4135±240	581±35	7.1
Sav^BPN^	Yes	174±21	101±27	1.7
Sav^Alc^	Yes	88±2	65±6	1.4
Sav^SbE^	Yes	38±3	−	−
Sav^ISP^	No	ND	ND	ND
Sav^AK1^	Yes	23±8	−	−
Sav^Ther^	Yes	13±2	−	−

a The parental subtilisin donating the sequence to each of the 6 regions is superscripted.

b The activity of the cell culture medium against substrate Suc-Phe-Ala-Ala-Phe-pNA. ND is not determined.

c The activity of the cell culture medium against substrate Suc-Ala-Ala-Pro-Phe-pNA. Cells marks with ‘–’ indicates no observable activity. ND is not determined.

d The ratio of the initial rates for the two substrates for each variant. Those marked with ‘–’ could not be calculated. ND is not determined.

The activity of reconstituted Sav towards AAPF was lower compared to that observed for FAAF, giving a FAAF:AAPF ratio of 7.1 (Table 1). This is in line with previous studies that have shown Sav has a preference for FAAF over AAPF [34]. Sav^SbE^, Sav^Ther^ and Sav^AK1^ had no observable activity towards the AAPF substrate suggesting a strong substrate preference for FAAF over AAPF (Table 1). However, both Sav^BPN^ and Sav^Alc^ displayed similar activities for both substrates, with FAAF:AAPF ratios of 1.7 and 1.4, respectively (Table 1). The level of activity of the rational fragment exchange variants towards the different substrates highlights the importance of the selected regions in defining the substrate specificity profile. For example, normally both Savinase and BPN' have a preference for Phe at the P4 position compared to Ala [35], [36] due to formation of a large hydrophobic binding pocket; this approaches parity when the BPN regions, including those critical to defining specificity of the P4 substrate position, are put in the context of the Sav backbone.

### Creation and screening Lib^R34^, a simple two region library

To assess the tolerance and impact of different sequence combinations and investigate if a combinatorial approach can generate variants with altered substrate profiles and activities greater than that produced by rational exchange above, libraries were constructed that allowed each region to sample one of the donor sequences in combination with any other donor sequence at the other regions. A small conservative library, called Lib^R34^, was created that combinatorially sampled all seven donor sequences at regions R3 and R4. These two regions show the highest sequence identity to Sav (Figure 2).

**Figure 6:**
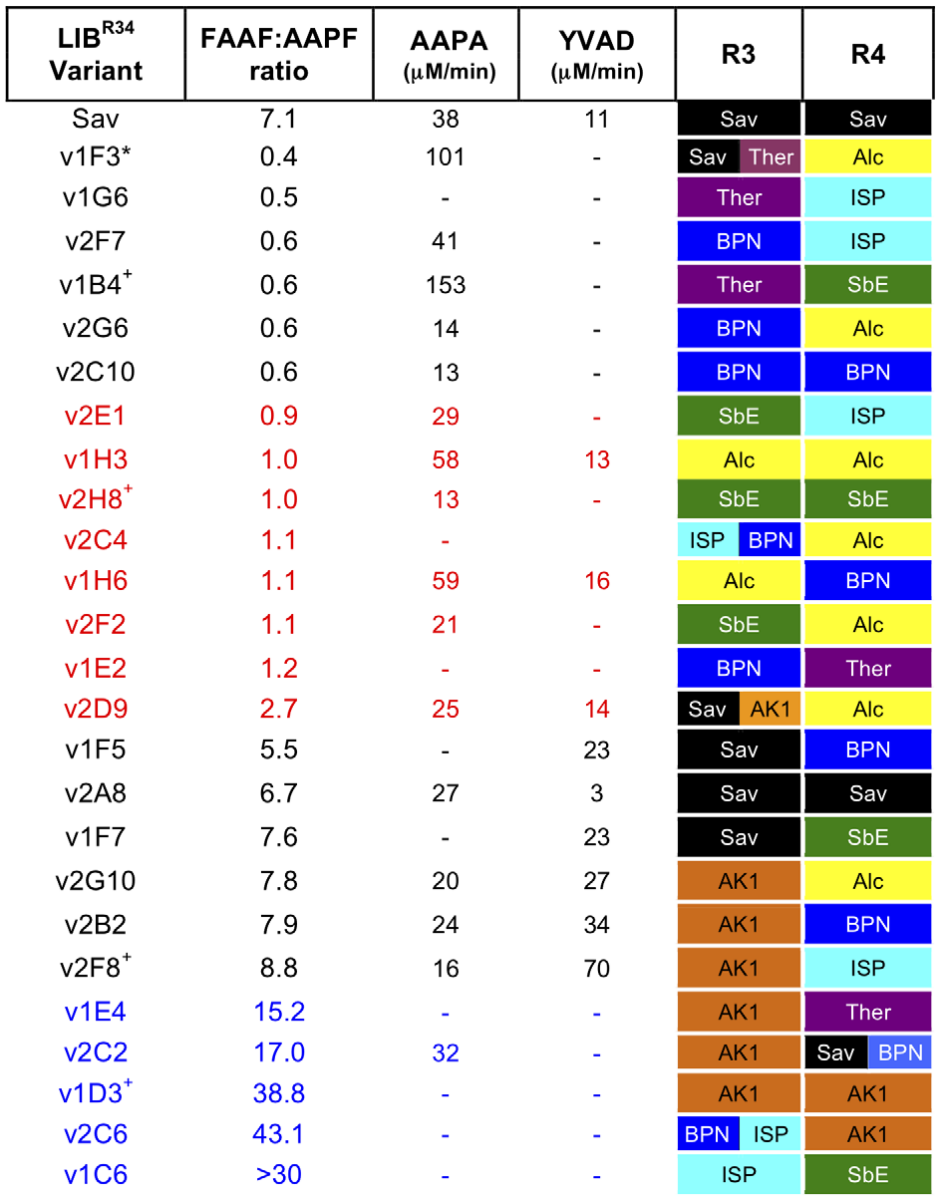
Characterisation of Lib^R34^ variants. Variants are blocked according to their FAAF:AAPF ratio as described in the main text. Variant v1F3 (labelled with *) has an additional N43L mutation. The sequence of R3 or R4 hybrid regions are described in Table S2. Data for variants labelled with + sharing the same sequence identified from different transformants is presented in Table S3.

Transformation of *B. subtilis* with Lib^R34^ resulted in ∼35% of all colonies producing clearing zones of varying sizes on casein-embedded LB agar plates indicating the production of an active protease. A representative selection is shown in Figure S1. Several variants that exhibited no protease activity were sequenced and most contained base pair insertions or deletions centred on R3, R4 and the linking oligonucleotide f4 that cause a frame shift (Table S1). This may in part be due to the size and quality of the oligonucleotides that encode R3 (73 bp), R4 (66 bp) and f4 (63 bp). Clones producing active variants were randomly chosen for sequencing and further analysis. Figure 6 shows a representation of the diversity of the sequences accepted at R3 and R4 that still allow the protein to fold, autoprocess and therefore become active. At both R3 and R4 all parental sequences are represented. R3 contains various hybrids with the bridging sequence being the conserved GlySerGly (Table S2). One variant, v2C2, also contained a hybrid sequence in R4, with 129-PS-130 bridging the Sav and BPN (Table S2). Several variants contained R3 and R4 sequences from parents with the lowest sequence identity to Sav. For example, variant v1D3 contains AK1 at both R3 and R4 (Figure 6) which results in the mutation of a total of 10 residues, 5 in R3 and 5 in R4, together with the incorporation of a potential disulphide bridge in R4. One variant, v2A8, reconstituted the Sav sequence.

The ability to cleave a wide range of protein substrates is one reason why Savinase has proved useful as a general protease in various applications. Therefore, broadening substrate specificity while retaining high activity would be a desirable feature to engineer into Savinase. Conversely, narrowing substrate specificity may be beneficial when more defined cleavage characteristics are required. As both R3 and R4 are involved in substrate binding, especially at the P4 position, the ability of library members to hydrolyse various pNA substrates was investigated. As well as FAAF and AAPF, two normally poor substrates for Sav were used, Succinyl-Tyr-Val-Ala-Asp-pNA (YVAD) and Succinyl-Ala-Ala-Pro-Ala-pNA (AAPA) to assess if any activity improvement on combinatorial sampling at R3 and R4 can be observed. Figure 6 shows the relative activities towards each of the peptide substrates for variants from Lib^R34^. The ratio of initial rates of FAAF and AAPF hydrolysis gave an indication of the substrate specificity independent of concentration of protease in each sample. The validation of the ratio approach is illustrated for variant v2A8, which reconstituted the Sav sequence and had a FAAF:AAPF ratio of 6.7, close to the value of 7.1 determined for Sav itself (Table 1). Furthermore, the observed activity of v2A8 towards FAAF and AAPF was 3502 µM/min and 527 µM/min, similar to that observed for Sav (Table 1). The robustness of the approach is further validated by the observation that variants with the same sequence identified from independently isolated transformants gave similar FAAF:AAPF ratios (Table S3).

As Figure 6 illustrates, the substrate preference of the variants depended on the sequence combinations at R3 and R4. Their FAAF:AAPF ratio falls into 4 groups: <1, ∼1, Sav-like and >10. Variants with a Sav-like FAAF:AAPF ratio had either AK1 or Sav sequences at R3 but displayed broader sequence sampling at R4. This is despite the Sav and AK1 sharing only 45% identity at R3. With respect to substrates AAPA and YVAD, another interesting trend emerges. Sav has a negligible ability to utilise YVAD (Sav and variant v2A8) but when R4 is replaced by SbE (v1F7) or BPN' (v1F5), the preference appeared to shift to YVAD, indicating that R4 can modulate the specificity together with R3. In contrast, the presence of AK1 at R3 together with BPN (v2B2) at R4 results in a ratio of AAPA to YVAD hydrolysis rate close to 1. Although these variants show a similar substrate profile on AAPF and FAAF to Sav, altering R4 can alter their ability to use AAPA and YVAD as substrate. All the variants with Sav-like ratio in Figure 6 retained a high degree of activity, with observed activity towards FAAF being >1500 µM/min. For example, the observed activity of v2B2 towards FAAF and AAPF was 3256 and 411 µM/min, respectively.

Variants were also identified in which substrate specificity was switched from FAAF to AAPF. Variants with a preference for AAPF over FAAF (ratio <1) have the Ther and BPN sequences predominating at R3 with R4 displaying more variety (Figure 6). The context of Ther sequence at R3 is critical as the rationally constructed Sav^Ther^ variant had an obvious preference FAAF over AAPF (Table 1). Even though substrate preference shifts from FAAF to AAPF, specificity towards AAPA over YVAD is maintained (Figure 6). Furthermore, the observed rates of AAPA hydrolysis were in some instances substantially higher than that of Sav (e.g. v1F3 and v1B4). The observed activities for many of these variants towards AAPF was also much higher than that displayed by Sav. For example, the rate of AAPF hydrolysis by v1B4 was 4749 µM/min, comparable to the rate of Sav towards its preferred substrate FAAF (Table 1). Variant 1F3 also displayed a ∼5 fold increase in observed activity towards AAPF (3014 µM/min) compared to Sav. This suggests that variants with a switched preference from FAAF to AAPF retain a high degree of activity, and significant levels of folded, functional protein can be produced.

The generation of variants with a high activity towards a broad range of substrates will have significant value as a general non-specific protease. Variants with a ratio close to 1 implies that the both FAAF and AAPF substrates are accepted with a similar preference. Unlike the other two ratio groupings, there appears to be a less predominance of certain sequences at R3 but SbE and Alc sequences at R3 only occur in this grouping (Figure 6). An AK1 hybrid sequence (v2D9) also show ratios lower than expected from the general trends observed for AK1 at R3. Thus, conversion of the AK1 sequence in to a hybrid at R3 can also modulate substrate preference. As observed earlier, if BPN is present at R3, there is a preference for AAPF over FAAF but variant v1E2 shows a similar preference for FAAF and AAPF when combined with Ther at R4. Those variants that display activity towards AAPA or YVAD have a preference for AAPA over YVAD. The majority of variants (5 out of the 8 in Figure 6) had an observed activity of >500 µM/min for each substrate suggesting that the variants retained a high degree of activity despite the now equal preference for FAAF and AAPF. For example, the observed activity of v1H3 was ∼1925 µM/min towards AAPF and FAAF; ∼3 fold higher for AAPF and ∼2 fold lower for FAAF compared to Sav (Table 1).

Variants with more defined substrate specificity may be beneficial when precise cleavage of a substrate is required. Variants with a FAAF:AAPF ratio greater than 9 have an overwhelming preference for FAAF. Two parental sequences predominate at R3; AK1 and to a lesser extent, ISP. It is clear that the sequence at R4 can influence substrate specificity when combined with AK1 at R3. The presence of AK1 at both R3 and R4 was observed in three independent variants (Figure 2 and Table S3) and all had ratios ∼35. This drops to a ratio of ∼15 when combined with Ther or a Sav-BPN hybrid at R4 and <9 if combined with ISP, BPN' and Alc. The first occurrence of ISP at R3 coincides with a high FAAF:AAPF ratio (>30). The ratio could not be correctly determined under the conditions used, as no measurable rate was observed for AAPF as substrate. Both the native ISP (v1C6) and the BPN-ISP hybrid (v2C6) have ratios over 30. With regards to substrates AAPA and YVAD, only v2C2 exhibited any measurable rate with AAPA. This variant also displayed the highest observed activity of any Lib^R34^ member towards FAAF, 4240 µM/min. However, 3 of the variants (v1E4, v2C6 and v1C6) had observed activities towards FAAF of <750 µM/min.

### Creation and screening of Lib^Rall^ with all six regions exchanged

**Figure 7:**
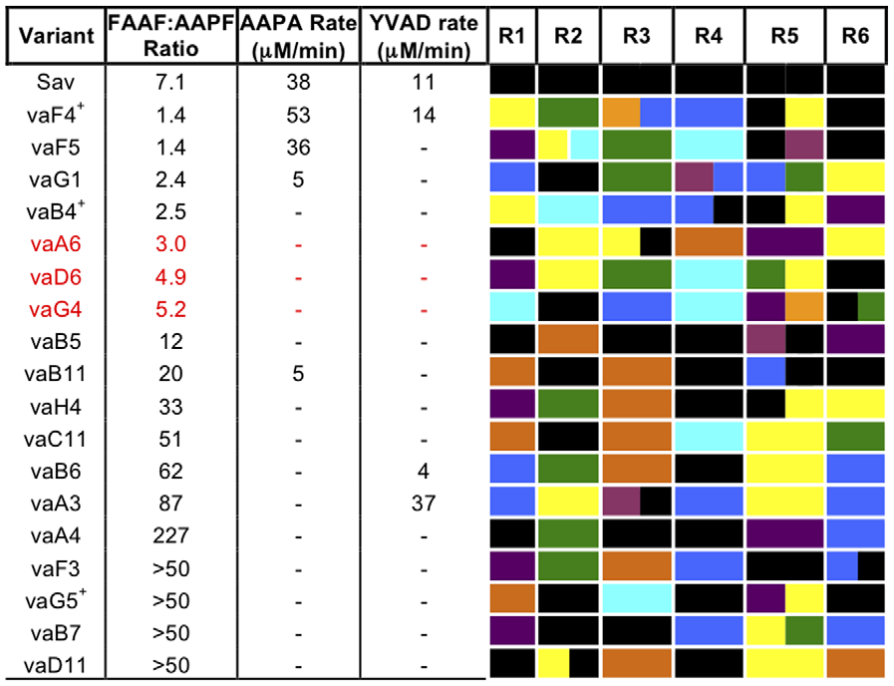
Characterisation of Lib^Rall^ variants. Variants are blocked according to their FAAF:AAPF ratio class as described in the main text. The colour code for regions R1 to R6 are: black, Sav; blue, BPN; yellow, Alc; green, SbE; orange, AK1; purple, Ther; cyan, ISP. Sequence of hybrid regions described in Table S4. Variants with additional mutations are labelled with a +: G206S (vaF4), N221I (vaB4), T23L (vaG5).

A larger library termed Lib^Rall^, which allowed combinatorial sampling at all six regions constituting almost 20% of Sav primary structure with sequences derived from all 7 parental sequences, was constructed. Approximately 5% of colonies exhibited protease activity as judged by the appearance of clearing zones on LB agar embedded with casein. Clones producing active variants were randomly chosen for sequencing and further analysis. A representative sample is shown in Figure S1. Sav-hybrid genes isolated from several colonies with no protease activity were sequenced and all contained nucleotide insertions or deletions that resulted in frame shifts. Most of the mutations arose in regions encoded by the oligonucleotides suggesting a quality issue with these chemically synthesised elements or that errors where introduced during gene assembly. Hybrid Sav genes were isolated from colonies that exhibited protease activity and sequenced, with a representative sample shown in Figure 7. The diversity of the combinations of sequences allowed to generate an active protease illustrates the plasticity of the Savinase fold.

As with Lib^R34^, the FAAF:AAPF activity ratio and activity towards YVAD and AAPA were used to judge general activity and substrate preference. The FAAF:AAPF ratio ranged from 1 to >200, with the library separating into three main groups; close to 1, Savinase-like (3–8) and high ratio (>10) (Figure 7). The variants with the lowest FAAF:AAPF ratios ranged from 1.35 to 3.00. No ratio below 1.0 was observed even though BPN was present at the R3 region, a major factor in Lib^R34^ that contributed to a low ratio. One variant, vaG4, contained BPN' at R3 and ISP at R4, the same as variant v2F7 from Lib^R34^, but the FAAF:AAPF ratio changes by a factor of 10 from 0.6 to 5.2 emphasising the role of the other four regions in determining the substrate specificity. Variant vaF5 had SbE at R3 and ISP at R4, the same as Lib^R34^ variant v2E1 and both had a FAAF:AAPF ratio close to 1 and had observable initial hydrolysis rates for AAPA. Variant vaD6 also contained SbE and ISP at R3 and R4, respectively but the FAAF:AAPF ratio increased to 4.9 (Figure 7). The only region to differ between vaF5 and vaD6 was R5; vaF5 contained a hybrid Sav-Ther sequence but vaD6 contained the SbE-Alc hybrid sequence that resulted in the insertion of four residues compared to Sav.

All the variants exhibiting a Savinase-like ratio contained a true Sav sequence at only one region, with vaA6 and vaG4 containing an additional Sav hybrid sequence (Figure 7). Variant vaG4 contained BPN and ISP at R3 and R4, the same as variant v2F7 from Lib^R34^ (Figure 6) but the FAAF:AAPF ratio jumps to 5.2 from 0.58, an increase of 9 fold.

Variants with an FAAF:AAPF ratio of >10 were predominant (Figure 7). Variant vaA4 is a good illustration that the combination of sequences rather than the absolute sequence at a particular region is important in defining the substrate profile. This variant contained Sav at three of the six regions including at R3 and R4 but the substrate ratio increased to 227, a factor of over 30 higher than Sav itself. The R2 region was donated by SbE as with variant vaF4 (ratio 1.4) and R5 was donated by Ther, also present in variant vaA6 (ratio 3.0), with the commonly observed BPN sequence occupying R6.

Very few variants displayed any activity towards AAPA or YVAD but this may be because of low levels of active, folded secreted protease produced by the cultures as overall activity towards FAAF and AAPF were also low. For example, 8 of the 18 variants in Figure 7 had an observed activity towards FAAF of <150 µM/min, a drop ∼30 fold compared to Sav (Table 1). Many of the Lib^Rall^ variants did retain a significant activity, with 5 of the 18 variants exhibiting an observed activity towards FAAF of >750 µmol/min. The Lib^Rall^ variant with the highest activity towards AAPF (820 µM/min) was vaF4 (Figure 6); the observed activity was higher than that measured for Sav (Table 1). This variant also exhibits noticeable activity towards YVAD and had FAAF:AAPF ratio close to 1. Although observed activity towards AAPA and YVAD was much less than that measured for FAAF and AAPF (1110 and 820 µM/min, respectively), such a protease that retains a significant activity on a wide range of substrates may have a utility as a general non-specific protease, which is ideal for many different biotechnological applications. As vaF4 had the highest observed activity of any Lib^Rall^ variant towards FAAF or AAPF, it suggests that catalytic performance has been enhanced or, more likely, higher levels of folded, functional protease are produced compared to other Lib^Rall^ members. The same could also be true for Lib^R34^ variants v1H3 and v1H6 as they also have a FAAF:AAPF ratio close to 1, have a high observed activity towards both FAAF and AAPF (∼1900 µmol/min for v1H3 and ∼1600 µmol/min for v1H6) and have an appreciable activity on both AAPA and YVAD (Figure 6).

Given that the effect of a single sequence region is dependent on its context in terms of the other regions exchanged and the Sav backbone, it is difficult to pinpoint exact mutations within a region that give rise to particular substrate profiles. This is exemplified by the nature of residue 104 in R3. Residue 104 is known to be important in defining the specificity of the S4 pocket [9], [36]. BPN, Alc and SbE all contain Tyr at 104 yet the FAAF:AAPF ratio for variants containing this mutation range from 0.6 (e.g. v2F7; Figure 6) to 5 (e.g. variant vaG4; Figure 7). Furthermore, Tyr104 in the context of BPN' contributes towards a preference for Phe at P4 compared to Ala [36]; in the context of the Sav backbone, in terms of the Sav^BPN^ variant, Tyr104 can contribute to a preference for Ala at P4 (Table 1). Both ISP and AK1 have larger residues (Met and Leu, respectively) at 104 compared to Sav (Val104) yet variants with ISP or AK1 at R3 generally have by far a greater preference for FAAF and not AAPF as may have been predicted. The caveat that also adds to the importance of the context of a mutation is variant v2D9 (Figure 6); this variant has a hybrid R3 sequence with the 104 residue donated by AK1 but has a FAAF:AAPF ratio of 2.7, less than Sav. Therefore, combinatorial fragment exchange allows mutations to sample different contexts, which may in turn modulate the effect of the mutation and thus functional output of the protein.

In conclusion, combinatorial fragment exchange allows multiple regions to be interchanged with sequence elements from sources with varying degrees of identities and in various combinations to produce new subtilisins variants not normally sampled by traditional rational protein engineering and directed evolution approaches. Using both rational and combinatorial fragment exchange, it was demonstrated that the Sav backbone was largely tolerant to exchanges in the 6 chosen regions demonstrating the structural plasticity of this particular subtilisin. The combinatorial component was especially powerful as it allowed a broader sampling of sequence space and the sequence/structure context a particular mutation experiences. This increases the chances of generating active variants with novel sequence combinations and hence functional properties, such as an altered substrate profile. As different distinct regions, such as loops, play a critical part in defining the properties of a protein this approach can be applied to different target proteins. The selected regions are already functionally rich and primed to lead to novel properties, so make the more lengthy library construction step worthwhile. The new variants could then provide the core scaffold containing key properties that forms the basis for further optimisation by random mutagenesis. Alternatively, knowledge on the contribution of each region to properties of a protein gleaned from analysis of combinatorial libraries could be used to rationally construct variants with region combinations best suited to a particular application.

## Materials and Methods

### Structural alignment

Prior to structural alignment a homology model of ISP was built as outlined in the Methods S1. The backbone atoms of the selected subtilisin structures, 1SVN (Sav)[37], 2ST1 (BPN') [38], 1SCJ (SbE)[39], 1SBC (Alc)[40], 1DBI (Ak.1)[31], 1THM (Ther)[41], were aligned using the core protein, Savinase, as the reference using the MagicFit option in the Swiss-PDBViewer [42]. The quality of the alignment was assessed by comparing the geometries of four conserved catalytic residues, D32, H64, N155 and S221 (numbering from PDB file 1SVN). A sequence alignment based on the structural alignment was generated that allowed the direct comparison between the amino sequence and the structural positioning of the amino acid residues. Six regions, designated R1, R2, R3, R4, R5 and R6 (Figure 2) were chosen for reasons outlined in the results and discussion section. The precise structural start and end points for each region were back-translated into the appropriate DNA sequence and flanked by a sequence encoding the constant Sav region either immediately before or after the region. The Sav sequence encoding the core of the protein that links each of the regions were double stranded DNA blocks generated by PCR or encoded by chemically synthesised oligonucleotides, as outlined in Figure 1.

### Construction of rational variants, Lib^R34^ and Lib^Rall^

Synthetic oligonucleotides were designed to encode the regions R1, R2, R3, R4, R5 and R6, from each donating subtilisin (Tables S5, S6, S7, S8, S9, S10). The segment of the oligonucleotide encoding each region was flanked by a DNA sequence at either the 5′ and/or 3′ end corresponding to the Sav amino acid sequence immediately before and/or immediately after each region. For both AK1 and Ther, additional mutations were included in the oligonucleotide sequence for R2 to encode incorporation of an aspartate residue equivalent to D58 and D57, respectively (Table S6).

The variants were constructed using the strategy outlined in Figure 1. In the case of the rational fragment exchange variants, regions R1 to R6 were donated from a single subtilisin. To construct Lib^Rall^, regions R1 to R6 were replaced and each region was allowed to sample all seven donor subtilisin sequences. To construct Lib^R34^, only regions R3 and R4 were combinatorially exchanged. The detailed description of rational variant and library construction is provided in the Methods S1.

### Screening for protease activity

*B. subtilis* PL1801 transformants exhibiting clearing zones (halos) on LB agar plates embedded with casein were picked and grown in 96 well microtitre plates containing 2TY liquid media supplemented with 6 mg/l chloramphenicol. The cells were grown for between 36 to 48 hrs. The library was subjected to a screen using various *p*-nitroanilides (pNA) peptide substrates (Bachem AG); Suc-Phe-Ala-Ala-Phe-pNA (FAAF), Suc-Ala-Ala-Pro-Phe-pNA (AAPF), Suc-Ala-Ala-Pro-Ala-pNA (AAPA) and Suc-Tyr-Val-Ala-Glu-pNA (YVAD). The pNA assay was performed in 96 well microtiter plates in 100 mM TrisHCl, pH 8.6, 0.0225% (v/v) Brij® 35 with various pNA substrates at a final concentration of 0.5 mg/ml and at various dilutions of cell culture medium. The cell culture medium dilutions ranged from 100 fold when measuring activity with substrates FAAF and AAPF to 20 fold when measuring activity with substrates AAPA and YVAD. The rate was determined by monitoring the increase in absorption at 405 nm. Initial rates were converted from absorbance to concentration using a molar absorbance coefficient of 9800 M^−1^cm^−1^ and extrapolated to zero culture dilution to allow comparison between different substrates and variants. To monitor substrate specificity, a ratio was calculated in which the observed rate of FAAF hydrolysis was compared directly to the AAPF hydrolysis rate for each analysed culture. The absolute rate was reported for the AAPA and YVAD as these were relatively poor substrates in comparison.

## Supporting Information

Methods S1 Homology model of ISP Construction of rational fragment exchange variants Construction of Lib^R34^ Construction of Lib^Rall^.
(DOC)

Figure S1 Identification of active Sav-hybrid variants by colony screening. Representative sample of colonies producing active protease from (A) Lib^R34^ and (B) Lib^Rall^ are shown. An active variant is indicated by the production of a clearing zone or halo around the *B. subtilis* colony due to digestion of casein embedded in the agar growth medium.
(PDF)

Table S1 Frameshift positions for inactive variants from library Lib^R34^.
(DOCX)

Table S2 Hybrid region sequences from library Lib^R34^.
(DOCX)

Table S3 Substrate specificity of Lib^R34^ variants with the same sequence but isolated from different transformants.
(DOCX)

Table S4 Hybrid sequences from Library Lib^Rall^.
(DOCX)

Table S5 Amino acid and oligonucleotide sequence for region R1.
(DOCX)

Table S6 Amino acid and oligonucleotide sequence for region R2.
(DOCX)

Table S7 Amino acid and oligonucleotide sequence for regions R3 and R4.
(DOCX)

Table S8 Amino acid and oligonucleotide sequence for region R5.
(DOCX)

Table S9 Amino acid and oligonucleotide sequence for region R6.
(DOCX)

Table S10 Sequences of linking oligonucleotides.
(DOCX)
