# Supplementary material for: Recombining Low Homology, Functionally Rich Regions of Bacterial Subtilisins by Combinatorial Fragment Exchange
Source: PLoS One. 2011 Sep 7;6(9):e24319. doi: 10.1371/journal.pone.0024319 (PMC3168465; doi:10.1371/journal.pone.0024319)
Supplement: Methods S1 — Homology model of ISP Construction of rational fragment exchange variants Construction of LibR34 Construction of LibRall. (DOC) [file pone.0024319.s001.doc]

Recombining low homology, functionally rich regions of bacterial subtilisins by combinatorial fragment exchange.

D. Dafydd Jones ^1,2^

^1^ School of Biosciences, Cardiff University, Cardiff UK.

^2^ Novozymes A/S, Bagsværd, Denmark.

Correspondence address: D. D. Jones, School of Biosciences, Main Building, Cardiff University, Cardiff, CF10 3AT, UK. Email: [jonesdd@cf.ac.uk](mailto:jonesdd@cf.ac.uk)

**Supporting Information**

**Supporting Methods.**

*Homology model of ISP*.

A homology model of ISP was generated using the SWISS-MODEL server with the PDB files 1YJA, 1YJB, 1YJC, 1SBH (all mutants of BPN’) and 1BFK (Subtilisin Carlsberg) as the templates. The sequence alignment was modified further manually to improve alignment, especially in regions where the sequence length varied and/or the homology was low. A new model based on the modified structure/sequence alignment was used as the starting template for SWISS-MODEL. Further rounds of energy minimisation using a version of the [GROMOS](http://www.igc.ethz.ch/gromos/) 43B1 force field were performed in order to further optimise the geometry of the protein. The structural quality of the resulting model was assessed using the WhatCheck program .

*Construction of rational fragment exchange variants*

Oligonucleotides were designed and synthesised to represent each of the 6 selected regions from the seven different subtilisins (Supporting Tables 1-5). The gene encoding Savinase (Sav) was fragmented as illustrated in Figure 1 in the main text. Four of the fragments (F1, F3, F5 and F7) were generated by PCR using Pwo polymerase (Roche). The remaining sections of the gene (f2, f4 and f6) were encoded by oligonucleotides (Supporting Table 6). The Sav gene fragments were spliced together in the presence of specific oligonucleotide combinations so as to generate each rational variant. PCR was performed using Pwo polymerase, containing 5 nM of fragments F1 (encoding residues –1 to 32), F3 (encoding residues 64 to 95), F5 (encoding residues 133 to153) and F7 (encoding residues 198 to 242) and 5 nM of oligonucleotides f2 (encoding residues 39 to 59), f4 (encoding residues 105 to 124) and f6 (encoding residues 175 to 187). Two different oligonucleotides comprised f2; the wild-type sequence and another coding the S49D mutation for Ther and AK. The 7 oligonucleotides encoding each of the six regions were added to a final concentration of 5 nM. Two terminal primers were added to a final concentration of 400 nM and the reassembly PCR was performed. The reassembly mixture was initially heated to 94°C for 2 min, which was followed by 10 cycles of 94°C for 15 seconds, 45°C for 30 seconds and 72°C for 1 minute. This was followed by a further 20 cycles but with 5 seconds added per cycle during the 72°C extension step. The resulting *circa* 715 bp fragment was purified by the Qiagen QIAquick Gel Extraction kit after agarose gel electrophoresis.

The vector pSX222 was linearised by PCR using the Expand Long™ Template PCR system (Roche), using primers that compliment the termini of the Savinase rational variant genes (5’-ccccatggtaccgattgcgcca-3’ and 5’-cgcagaagcggcaacgcgttaatc-3’). The rational Savinase variant genes were ligated into the pSX222 plasmid by the process of multimerisation, using the Expand Long™ Template PCR system (Roche) with 0.5 pmol of variant insert gene, 0.03 pmol of linear pSX222, 1.6 mM dNTP, 2.5 units of DNA polymerase mix. PCR was performed by initially heating the reaction mixture to 94°C for 1 min followed by 10 rounds of Cycle 1 (94°C for 10 sec, 55°C for 30 sec, 68°C for 5 min) and 15 to 35 rounds of Cycle 2 (94°C 10 sec, 55°C for 30 sec, 68°C for 10 min). Multimerisation was confirmed by agarose gel electrophoresis and was subsequently used to transform *B. subtilis* PL1801. The transformants were screened for their ability to survive on LB agar plates supplemented with 6 μg/ml chloramphenicol and to produce clearing zone (or halos) by digestion of the media-embedded casein. Those colonies exhibiting protease activity were subject to PCR using primers that flank the mature version Savinase and the resulting PCR products to confirm the sequence of the variant. As colonies originating from the rational variant with all regions replaced by ISP exhibited no protease activity, a number of colonies were subjected to PCR to confirm the correct identity of the rational variant.

# Construction of Lib^R34^

The Savinase gene was fragmented by PCR to create two sections; the first product encompassed fragments F1, f2 and F3 (equivalent to residues –1 to 96) and the second product encompassed fragments F5, f6 and F7 (equivalent to residues 133 to 242). Oligonucleotide f4 (Supporting Table 6) was used to encode the region between R3 and R4. Each individual fragment is outlined in Figure 1 in the main text.

The fragments generated above were spliced together in the presence of the oligonucleotides encoding the donating R3 and R4 regions (Supporting Table 3) thereby regenerating full-length core protein genes with variation in at R3 and R4. The splice PCR was performed as described for the creation of the rational variants except for the following. A pool of either R3 or R4 encoding oligonucleotides was created in which all the oligonucleotides were present in equal amounts. The R3 pool and R4 pool of oligonucleotides were each added individually to the reaction mixture to a final concentration of 5 nM. The resulting *circa* 715 bp R3-R4 library fragments were gel-purified and ligated into pSX222 by multimerisation as described above, thus generating the library Lib^R34^. Multimerisation, transformation and selection for active clones were performed as outlined above.

# Construction of Lib^Rall^

The Lib^Rall^ library was created in a similar manner to that of the rational variants except that combinatorial sampling of all parent sequences was allowed at each of the 6 regions. The library was constructed in two steps. Firstly, diversity was generated at R1 using the fragment F1 (0.5 nM) as the template, the Sav gene 5’ terminal sense primer (5’- tggcgcaatcggtaccatgggg-3’) and an equimolar concentration of each R1 oligonucleotide (Supporting Table 1) (total concentration 0.4 μM) as the antisense primer in a standard PCR reaction using Pwo polymerase. The *circa* 180 bp fragment was gel purified after agarose gel electrophoresis; this new fragment is termed F1R1. Secondly diversity was generated at the R2 position as follows. Two different oligonucleotides that link regions R1 and R2 (Supporting Table 6) were used as sense primers; the molar ratio of the ‘no Asp’ and ‘inc Asp’ oligonucleotides was 5:2 so as to represent the observation that two parental regions (AK1 and Ther) out of seven require the S49D mutation to fully form a calcium binding site. An oligonucleotide (5’- taggactttaacagcgtatagctcagcg-3’) was used as the antisense primer together with fragment F3 (5 nM) and an equimolar concentration of each R2 oligonucleotide (total concentration 5 nM), which acted as the template, were used in a standard PCR reaction using Pwo polymerase. The sub-assembly of this section of the gene library resulted in a PCR product of 175 bp and was extracted from agarose gel after electrophoresis; this fragment is termed F3R2. Thirdly, two fragments F5 and F7 were identical to those used in the construction of the rational variants. Fragments F1R1, F3R2, F5 and F6, together with oligonucleotides encoding Savinase residues f4 and f6 were spliced together in the presence of R3, R4, R5 and R6 oligonucleotides by PCR to generate full length core protein gene with variation at the all R-regions. PCR was performed as described for the construction of the rational variants. The reaction mixture contained 5 nM of each Savinase-encoding fragment and oligonucleotide together with 0.8 μM of terminal primers. A pool of R3, R4, R5 or R6 encoding oligonucleotides was created in which all the oligonucleotides representing that region were present in equal amounts. The R3, R4, R5 and R6 pools were added individually to the reaction mixture to a final concentration of 5 nM. The resulting *circa* 715 bp fragment was extracted from agarose gel after electrophoresis. The subsequent ligation into pSX222 by multimerisation followed by transformation into the host cell and selection for clones expressing active protease were performed as described previously.

**Supporting References**

1. Arnold K, Bordoli L, Kopp J, Schwede T (2006) The SWISS-MODEL workspace: a web-based environment for protein structure homology modelling. Bioinformatics 22: 195-201.

2. Kiefer F, Arnold K, Kunzli M, Bordoli L, Schwede T (2009) The SWISS-MODEL Repository and associated resources. Nucleic Acids Res 37: D387-392.

3. Hooft RW, Vriend G, Sander C, Abola EE (1996) Errors in protein structures. Nature 381: 272.
