# Supplementary figures and images for: Recombining Low Homology, Functionally Rich Regions of Bacterial Subtilisins by Combinatorial Fragment Exchange
Source: PLoS One. 2011 Sep 7;6(9):e24319. doi: 10.1371/journal.pone.0024319 (PMC3168465; doi:10.1371/journal.pone.0024319)

**A**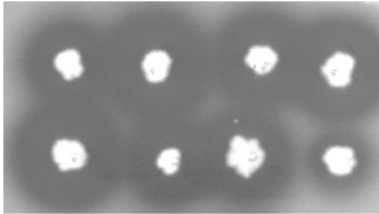**B**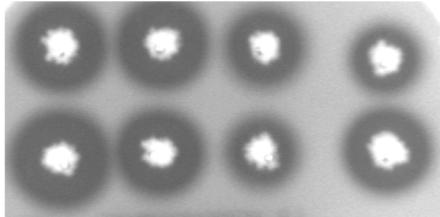

Supplement: Figure S1 — Identification of active Sav-hybrid variants by colony screening. Representative sample of colonies producing active protease from (A) LibR34 and (B) LibRall are shown. An active variant is indicated by the production of a clearing zone or halo around the B. subtilis colony due to digestion of casein embedded in the agar growth medium. (PDF) [file pone.0024319.s002.pdf]
