# Supplementary material for: Recombining Low Homology, Functionally Rich Regions of Bacterial Subtilisins by Combinatorial Fragment Exchange
Source: PLoS One. 2011 Sep 7;6(9):e24319. doi: 10.1371/journal.pone.0024319 (PMC3168465; doi:10.1371/journal.pone.0024319)
Supplement: Table S1 — Frameshift positions for inactive variants from library LibR34. (DOCX) [file pone.0024319.s003.docx]

**Supporting Table 1. Frameshift positions for inactive variants from library Lib^R34^.**

| **Inactive variant** | **Frame shift mutation encoded in oligonucleotide ^a^** | **Region** |
| --- | --- | --- |
| 873 | TCTAGCGGTTCAGGTTCGTATagct | R3 |
|  | TCTAGCGGTTCAGGTTCG-ATagct |  |
| 872 | gctgagctata-gctgttaaagtcCTAGGGG | R3 |
|  | gctgagctata**c**gctgttaaagtcCTAGGGG |  |
| 871 | GCGGATGGTTCAGGTCAATATagctcgattgcccaaggattg | R3 |
|  | gcggatggttcag----atatAGCTCGATTGCCCAAGGATTG |  |
| 869 | ggcatgcac-gttgctaatttgAGTTTA | R4 |
|  | ggcatgcac**c**gttgctaatttgAGTTTA |  |
| 885 | ggattgg-aatgggcagggaacaatggcat | f4 (R3-R4 linker) |
|  | ggattgg**g**aatgggcagggaacaatggcat |  |
| 884 | ggattggaatgggcagggaacaatggcat | f4 (R3-R4 linker) |
|  | ggattggaatgggcaggga**-**caatggcat |  |

^a^ the requested sequence of the oligonucleotide is in red and actual sequence in inactive variant is below in black. Sequences in capitals represent exchange regions and those in lower case linking sections between regions.
