# Supplementary material for: Recombining Low Homology, Functionally Rich Regions of Bacterial Subtilisins by Combinatorial Fragment Exchange
Source: PLoS One. 2011 Sep 7;6(9):e24319. doi: 10.1371/journal.pone.0024319 (PMC3168465; doi:10.1371/journal.pone.0024319)
Supplement: Table S2 — Hybrid region sequences from library LibR34. (DOCX) [file pone.0024319.s004.docx]

**Supporting Table 2. Hybrid region sequences from library Lib^R34^.**

| **LIB^R34^ Variant** | **R3** | **R4** |
| --- | --- | --- |
| v1F3 | LGASGSG**TW** |  |
|  | Sav-Ther |  |
| v2C4 | LSGDGSG**QY** |  |
|  | ISP-BPN |  |
| v2D9 | LGASGSG**TL** |  |
|  | Sav-AK1 | Alc |
| v2C2 |  | SLGSPS**GS** |
|  |  | Sav-BPN |
| v2C6 | **LGAD**GSG**EM** |  |
|  | BPN-ISP |  |
