# Supplementary material for: Recombining Low Homology, Functionally Rich Regions of Bacterial Subtilisins by Combinatorial Fragment Exchange
Source: PLoS One. 2011 Sep 7;6(9):e24319. doi: 10.1371/journal.pone.0024319 (PMC3168465; doi:10.1371/journal.pone.0024319)
Supplement: Table S3 — Substrate specificity of LibR34 variants with the same sequence but isolated from different transformants. (DOCX) [file pone.0024319.s005.docx]

**Supporting Table 3. Substrate specificity of Lib^R34^ variants with the same sequence but isolated from different transformants.**

| **Variant** | **FAAF:AAPF** | **R3** | **R4** |
| --- | --- | --- | --- |
| v1B4^a^ | 0.6 | Ther | SbE |
| v1C5 | 0.6 |  |  |
| v2H8^a^ | 1.0 | SbE | SbE |
| v1B5 | 1.0 |  |  |
| v2F8^a^ | 8.8 | AK1 | ISP |
| v2C8 | 9.6 |  |  |
| v1D3^a^ | 38.8 | AK1 | AK1 |
| v2B3 | 34.0 |  |  |
| v2E2 | 33.5 |  |  |

a, variants reported in Table 3 in the main manuscript.
