# Supplementary material for: Recombining Low Homology, Functionally Rich Regions of Bacterial Subtilisins by Combinatorial Fragment Exchange
Source: PLoS One. 2011 Sep 7;6(9):e24319. doi: 10.1371/journal.pone.0024319 (PMC3168465; doi:10.1371/journal.pone.0024319)
Supplement: Table S4 — Hybrid sequences from Library LibRall. (DOCX) [file pone.0024319.s006.docx]

**Supporting Table 4. Hybrid sequences from Library Lib^Rall^.**

| **LIB^R34^ Variant** | **R3^a^** | **R4^a^** | **R5^a^** | **R6^a^** |
| --- | --- | --- | --- | --- |
| vaF4 | **LDRN**GSG**QY**  AK1-BPN |  | **GNSGAGSIS**YPA**KYDSV**  Sav-Alc |  |
| vaF5 |  |  | **GNSGAGSIS**YPA**YYSNA**  Sav-Ther |  |
| vaG1 |  | **SLGGTSGS**  Ther-BPN | **GNEGTSGSSS**TVGYP**AKYPST**  BPN-SbE |  |
| vaB4 |  | **SLGG**PS**PS**  BPN-Sav | **GNSGAGSIS**YPA**KYDSV**  Sav-Alc |  |
| vaA6 | **LNSS**GSG**SV**  Alc-Sav |  |  |  |
| vaD6 |  |  | **GNEGS**SGST**NTIGYPAKYDSV**  SbE-Alc |  |
| vaG4 |  |  | **GNAGNTAPNYPAYYS**N**V**  Ther-AK1 |  |
| vaB5 |  |  | **GNAGNTAPN**YPA**RYANA**  Ther-Sav |  |
| vaB11 |  |  | **GNEGTS**GS**ISYPARYANA**  BPN-Sav |  |
| vaH4 |  |  | **GNSGAGSIS**YPA**KYDSV**  Sav-Alc |  |
| vaA3 | **LDNS**GSG**SV**  Ther-Sav |  |  |  |
| vaF3 |  |  |  | **SFSSV**G**AGLD**  BPN-Sav |
| vaG5 |  |  | **GNAGNTAPN**YPA**KYDSV**  Ther-Alc |  |
| vaB7 |  |  | **GNSGN**SGST**STVGYPAKYPST**  Alc-SbE |  |

**^a^** for all hybrid sequences, the bold represent the sequence section unique to one of the donating subtilisin and normal text represents the common sequence section that links the two different sequences.
