# Supplementary material for: Recombining Low Homology, Functionally Rich Regions of Bacterial Subtilisins by Combinatorial Fragment Exchange
Source: PLoS One. 2011 Sep 7;6(9):e24319. doi: 10.1371/journal.pone.0024319 (PMC3168465; doi:10.1371/journal.pone.0024319)
Supplement: Table S5 — Amino acid and oligonucleotide sequence for region R1. (DOCX) [file pone.0024319.s007.docx]

**Supporting Table 5. Amino acid and oligonucleotide sequence for region R1**

| **Protein** | **Protein/**  **Oligonucleotide sequence (antisense)** |
| --- | --- |
| Sav | **TGIST**  5’accacgaatattaagatctggatgCGTTGAtATGCCTGTatcgaggacagcaacttttacacca |
| BPN'/SbE | **SGIDSS**  5’accacgaatattaagatctggatgTGAGCTATCtATGCCTGAatcgaggacagcaacttttacacca |
| Alc | **TGIQAS**  5’accacgaatattaagatctggatgTGATGCTTGtATGCCTGTatcgaggacagcaacttttacacca |
| ISP | **TGCQVD**  5’accacgaatattaagatctggatgATCGACTTGGCAGCCTGTatcgaggacagcaacttttacacca |
| AK1 | **TGVDYT**  5’accacgaatattaagatctggatgCGTATAATCAACGCCTGTatcgaggacagcaacttttacacca |
| Ther | **TGVQSD**  5’accacgaatattaagatctggatgATTTGATTGAACGCCTGTatcgaggacagcaacttttacacca |
