# Supplementary material for: Recombining Low Homology, Functionally Rich Regions of Bacterial Subtilisins by Combinatorial Fragment Exchange
Source: PLoS One. 2011 Sep 7;6(9):e24319. doi: 10.1371/journal.pone.0024319 (PMC3168465; doi:10.1371/journal.pone.0024319)
Supplement: Table S6 — Amino acid and oligonucleotide sequence for region R2. (DOCX) [file pone.0024319.s008.docx]

**Supporting Table 6. Amino acid and oligonucleotide sequence for region R2**

| **Protein** | **Protein/**  **Oligonucleotide sequence** |
| --- | --- |
| Sav | **VPGEPSTQDGNG**  5’cgtggtggcgcaagctttGTACCAGGGGAACCGTCAACACAAGATGGGAATGGGcatggcacgcatgtggccgg |
| BPN' | **VPSETNPFQDNNS**  5’cgtggtggcgcaagctttGTACCAAGCGAAACAAATCCGTTTCAAGATAACAATTCAcatggcacgcatgtggccggg |
| Alc | **VAGEAYNTDGNG**  5’attcgtggtggcgcaagctttGTaGCGGGgGAAGCGTATAATACAGATGGGAATGGGcatggcacgcatgtggc |
| SbE | **VPSETNPYQDGSS**  5’attcgtggtggcgcaagctttGTaCCaAGCGAAACAAATCCgTATCAAGATGGGAGCTCAcatggcacgcatgtggccggg |
| ISP | **TTDYGGDETNFSDNNG**  5’attcgtggtggcgcaagctttACAACGGATTATGGgGGAGATGAAACAAATTTTTCAGATAACAATGGGcatggcacgcatgtggccggg |
| AK1 | **D VDNDYDPMDLNN**  5’attcgtggtggcgcaGACtttGTaGATAATGATTATGATCCgATGGATTTAAATAACcatggcacgcatgtggccggg |
| Ther | **D VDNDSTPQNGN**  5’attcgtggtggcgcaGACtttGTaGATAATGATTCAACACCgCAAAATGGGAATGGGcatggcacgcatgtggc |
