# Supplementary material for: Recombining Low Homology, Functionally Rich Regions of Bacterial Subtilisins by Combinatorial Fragment Exchange
Source: PLoS One. 2011 Sep 7;6(9):e24319. doi: 10.1371/journal.pone.0024319 (PMC3168465; doi:10.1371/journal.pone.0024319)
Supplement: Table S7 — Amino acid and oligonucleotide sequence for regions R3 and R4. (DOCX) [file pone.0024319.s009.docx]

| **R3** |  |
| --- | --- |
| **Protein** | **Protein/**  **Oligonucleotide sequence (antisense)** |
| Sav | **LGASGSGSV**  5’caatccttgggcaatcgagctgaccgAaCCtGAaCCgctcGCcCCtAGgactttaacagcgtatagctcagc |
| BPN | **LGADGSGQY**  5’caatccttgggcaatcgagctATATTGaCCtGAaCCATCcGCcCCtAGgactttaacagcgtatagctcagc |
| Alc | **LNSSGSGSY**  5’caatccttgggcaatcgagctATACGAaCCtGAaCCgctAGAGTTtAGgactttaacagcgtatagctcagc |
| SbE | **LDSTGSGQY**  5’caatccttgggcaatcgagctATATTGaCCtGAaCCTGTAGAATCtAGgactttaacagcgtatagctcagc |
| ISP | **LSGDGSGEM**  5’caatccttgggcaatcgagctCATTTCaCCtGAaCCATCGCCTGAtAGgactttaacagcgtatagctcagc |
| AK1 | **LDRNGSGTL**  5’caatccttgggcaatcgagctGAGTGTaCCtGAaCCATTTCTATCtAGgactttaacagcgtatagctcagc |
| Ther | **LDNSGSGTW**  5’caatccttgggcaatcgagctCCATGTaCCtGAaCCgctGTTATCtAGgactttaacagcgtatagctcagc |
|  |  |
| **R4** |  |
| **Protein** | **Oligonucleotide sequence** |
| Sav | **SLGSPSPS**  5’ggcatgcacgttgctaatttgAGTTTAGGAAGCCCtTCTCCaAGTgccacactcgagcaagctgtt |
| BPN | **SLGGPSGS**  5’ggcatgcacgttgctaatttgAGTTTAGGAGGCCCtTCTGGAAGTgccacactcgagcaagctgtt |
| Alc | **SLGGASGS**  5’ggcatgcacgttgctaatttgAGTTTAGGAGGAGCGTCTGGAAGTgccacactcgagcaagctgtt |
| SbE | **SLGGPTGS**  5’ggcatgcacgttgctaatttgAGTTTAGGAGGACCtACAGGAAGTgccacactcgagcaagctgtt |
| ISP | **SLGGPTDS**  5’ggcatgcacgttgctaatttgAGTTTAGGAGGACCtACAGATAGTgccacactcgagcaagctgtt |
| AK1 | **SLGCDCHT**  5’ggcatgcacgttgctaatttgAGTTTAGGATGCGATTGTCATACAgccacactcgagcaagctgtt |
| Ther | **SLGGTVGN**  5’ggcatgcacgttgctaatttgAGTTTAGGAGGAACAGTTGGAAATgccacactcgagcaagctgtt |

**Supporting Table 7. Amino acid and oligonucleotide sequence for regions R3 and R4**
