# Supplementary material for: Recombining Low Homology, Functionally Rich Regions of Bacterial Subtilisins by Combinatorial Fragment Exchange
Source: PLoS One. 2011 Sep 7;6(9):e24319. doi: 10.1371/journal.pone.0024319 (PMC3168465; doi:10.1371/journal.pone.0024319)
Supplement: Table S8 — Amino acid and oligonucleotide sequence for region R5. (DOCX) [file pone.0024319.s010.docx]

**Supporting Table 8. Amino acid and oligonucleotide sequence for region R5**

| **Protein** | **Protein/**  **Oligonucleotide sequence** |
| --- | --- |
| Sav | **GNSGAGSISYPARYANA**  5'ATAGCGCGCCGGATAGCTGATTGAGCCTGCACCTGAATTCCCagatgccgctacaacaagaacgcctct |
|  | 5'GGCTCAATCAGCTATCCGGCGCGCTATGCGAACGCAatggcagtcggagctactgatcaaaac |
| BPN' | **GNEGTSGSSSTVGYPGKYPSV**  5'ATAGCCAACTGTAGAGCTTGAGCCGCTTGTACCTTCATTCCCagatgccgctacaacaagaacgcctct |
|  | 5'GGCTCAAGCTCTACAGTTGGCTATCCGGGAAAATATCCGAGCGTTatggcagtcggagctactgatcaaaac |
| Alc | **GNSGNSGSTNTIGYPAKYDSV**  5'ATAGCCGATTGTGTTCGTTGAGCCGCTATTACCTGAATTCCCagatgccgctacaacaagaacgcctct |
|  | 5'AGCGGCTCAACGAACACAATCGGCTATCCGGCGAAATATGATAGCGTTatggcagtcggagctactgatcaaaac |
| SbE | **GNEGSSGSTSTVGYPAKYPST**  5'CGGATAGCCAACTGTAGACGTTGAGCCGCTTGAACCTTCATTCCCagatgccgctacaacaagaacgcctct |
|  | 5'GGCTCAACGTCTACAGTTGGCTATCCGGCGAAATATCCGAGCACAatggcagtcggagctactgatcaaaac |
| ISP | **GNEGDGREDTNEFAYPAAYNEV**  5'ATATGCAAATTCGTTCGTGTCCTCTCTACCATCACCTTCATTCCCagatgccgctacaacaagaacgcctct |
|  | 5'AGAGAGGACACGAACGAATTTGCATATCCGGCGGCGTATAATGAAGTTatggcagtcggagctactgatcaaaac |
| AK1 | **GNNGSSTTFEPASYENV**  5'TTCATAGCTCGCCGGTTCAAACGTTGTGCTTGAACCGTTATTCCCagatgccgctacaacaagaacgcctct |
|  | 5'ACAACGTTTGAACCGGCGAGCTATGAAAACGTTatggcagtcggagctactgatcaaaac |
| Ther | **GNAGNTAPNYPAYYSNA**  5'GCTATAGTACGCCGGATAGTTCGGCGCTGTATTACCAGCATTCCCagatgccgctacaacaagaacgcctct |
|  | 5'ACAGCGCCGAACTATCCGGCGTACTATAGCAACGCAatggcagtcggagctactgatcaaaac |
