# Supplementary material for: Recombining Low Homology, Functionally Rich Regions of Bacterial Subtilisins by Combinatorial Fragment Exchange
Source: PLoS One. 2011 Sep 7;6(9):e24319. doi: 10.1371/journal.pone.0024319 (PMC3168465; doi:10.1371/journal.pone.0024319)
Supplement: Table S9 — Amino acid and oligonucleotide sequence for region R6. (DOCX) [file pone.0024319.s011.docx]

**Supporting Table 9. Amino acid and oligonucleotide sequence for region R6**

| **Protein** | **Protein/**  **Oligonucleotide sequence** |
| --- | --- |
| Sav | **SFSQYGAGLD**  5’gatcaaaacaacaaccgcgctAGCTTTTCACAGTATGGCGCAGGCCTTGACattgtcgcacccggggtaaac |
| BPN' | **SFSSVGPELD**  5’gatcaaaacaacaaccgcgctAGCTTTTCAAGCGTTGGCCCGGAACTTGACattgtcgcacccggggtaaac |
| Alc | **SFSSVGAELE**  5’gatcaaaacaacaaccgcgctAGCTTTTCAAGCGTTGGCGCAGAACTTGAGattgtcgcacccggggtaaac |
| SbE | **SFSSAGSELD**  5’gatcaaaacaacaaccgcgctAGCTTTTCAAGCGCAGGCTCAGAACTTGACattgtcgcacccggggtaaac |
| ISP | **DFTNTNEEID**  5’gatcaaaacaacaaccgcgctGATTTTACAAATACGAACGAGGAAATTGACattgtcgcacccggggtaaac |
| AK1 | **SFSNYGTWVD**  5’gatcaaaacaacaaccgcgctAGCTTTTCAAATTATGGCACATGGGTTGACattgtcgcacccggggtaaac |
| Ther | **SFSTYGSWVD**  5’gatcaaaacaacaaccgcgctAGCTTTTCAACATATGGCAGCTGGGTTGACattgtcgcacccggggtaaac |
