# Supplementary material for: Recombining Low Homology, Functionally Rich Regions of Bacterial Subtilisins by Combinatorial Fragment Exchange
Source: PLoS One. 2011 Sep 7;6(9):e24319. doi: 10.1371/journal.pone.0024319 (PMC3168465; doi:10.1371/journal.pone.0024319)
Supplement: Table S10 — Sequences of linking oligonucleotides. (DOCX) [file pone.0024319.s012.docx]

**Supporting Table 10. Sequences of linking oligonucleotides**

| **Fragment** | **Regions linked** | **Oligonucleotide sequence** |
| --- | --- | --- |
| f2 | R1 and R2  (no Asp) | 3’gtaggtctagaattataagcacca **R1 end**  **5’CATCCAGATCTTAATATTCGTGGTGGCGCAAGCTTT 3’** Wild-type  **R2 end** 5’attcgtggtggcgcaagcttt |
| f2 | R1 and R2  (inc Asp) | 3’gtaggtctagaattataagcacca **R1 end**  **5’CATCCAGATCTTAATATTCGTGGTGGCGCAgacTTT** inc Asp  **R2 end**  5’attcgtggtggcgca**GAC**ttt |
| f4 | R3 and R4 | 3’tcgagctaacgggttcctaac **R3 end**  5’agctcgattgcccaaggattggaatgggcagggaacaatggcatgcacgttgctaatttgagt  **R4 end** 5’ggcatgcacgttgctaatttg |
| f6 | R5 and R6 | 3’taccgtcagcctcgatgactagttttg **R5 end**  **5’atggcagtcggagctactgatcaaaacaacaaccgcgct**  **R6 end** 5’gatcaaaacaacaaccgcgct |
